# Supplementary figures and images for: Pericytes control vascular stability and auditory spiral ganglion neuron survival
Source: eLife. 2023 Jan 31;12:e83486. doi: 10.7554/eLife.83486 (PMC9940910; doi:10.7554/eLife.83486)

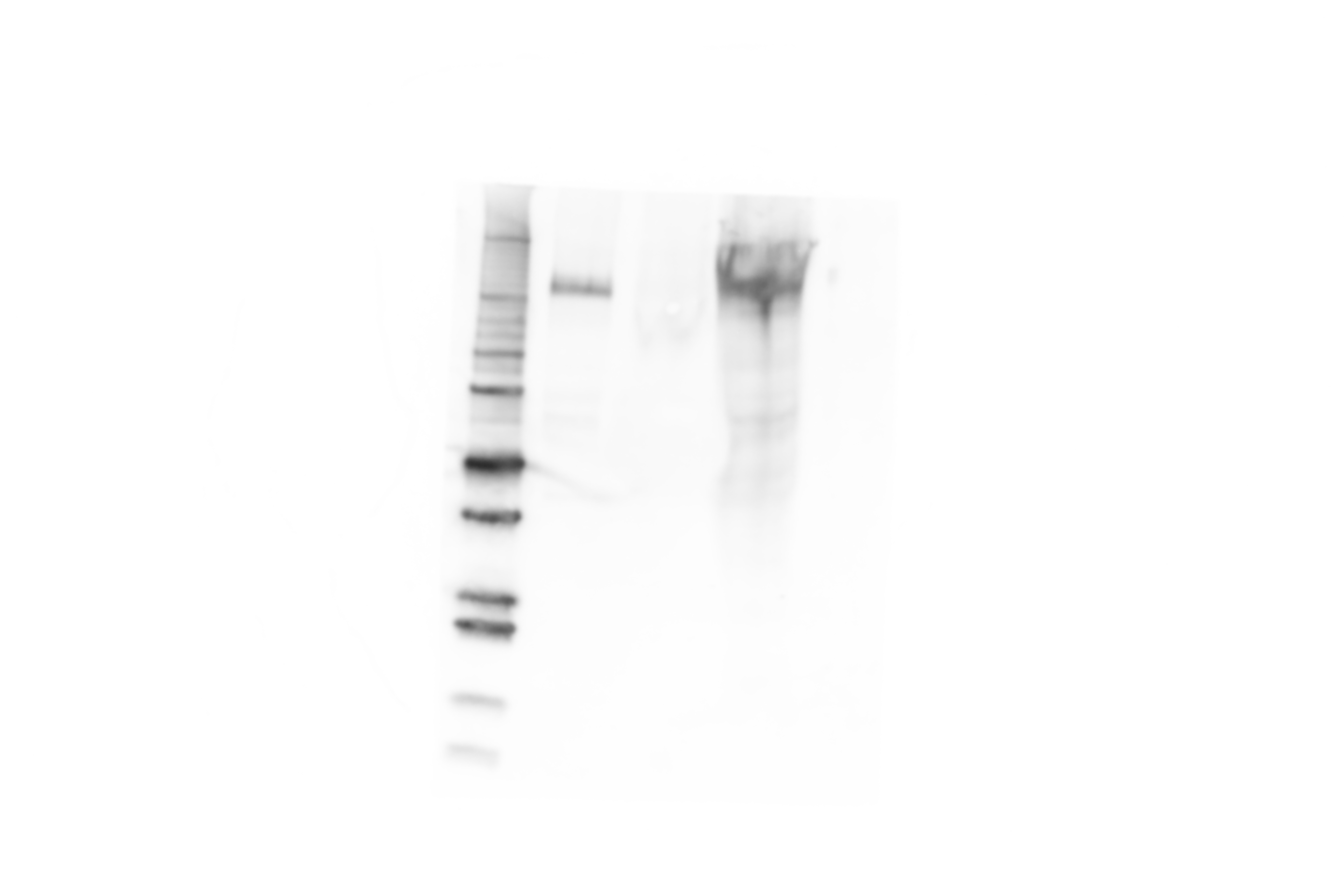

Supplement: Figure 7—source data 1. [file elife-83486-fig7-data1.zip › Figure 7-source data 1/Figure 7F-PDGFRb blot original.jpg]

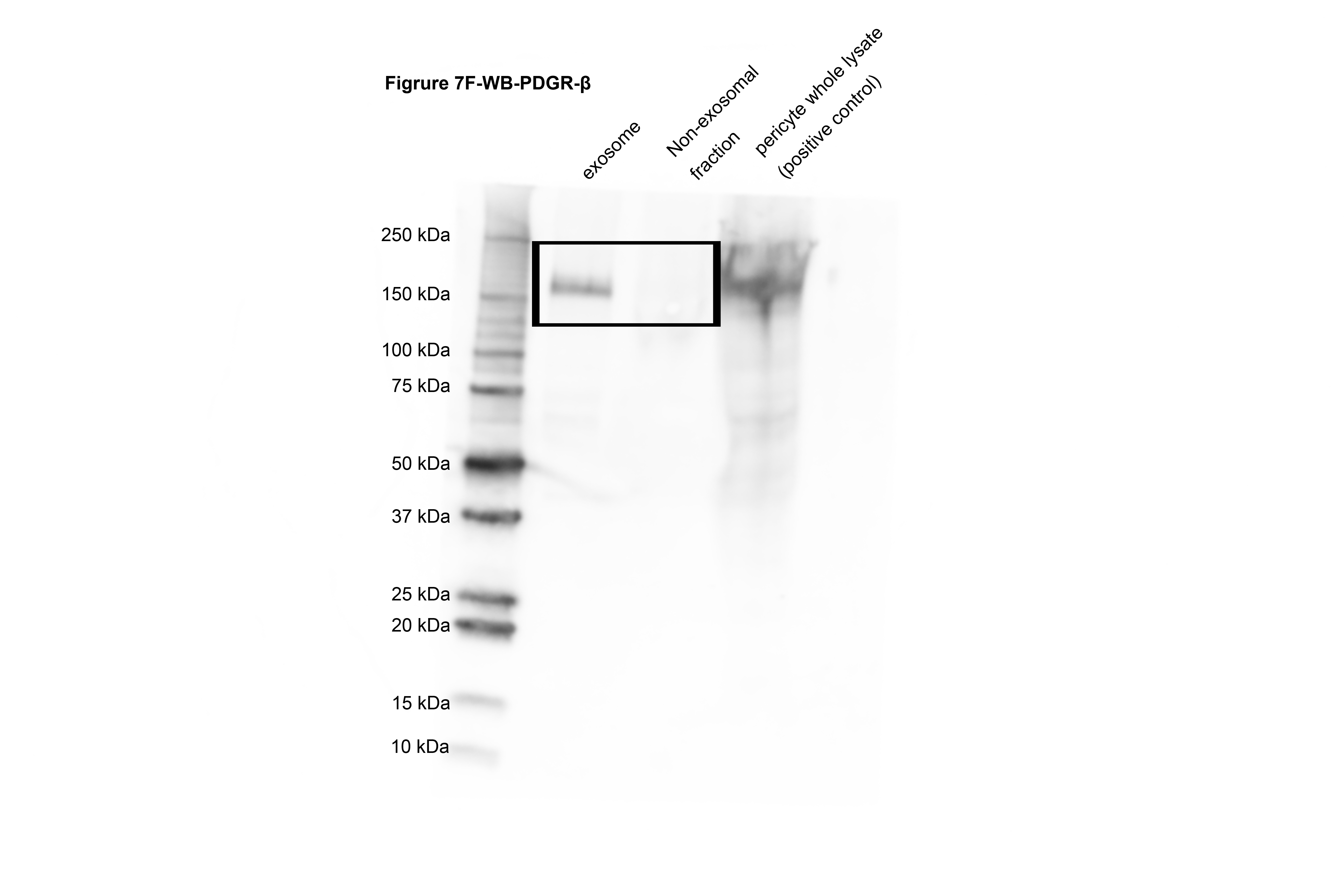

Supplement: Figure 7—source data 1. [file elife-83486-fig7-data1.zip › Figure 7-source data 1/Figure 7F-PDGFRb blot labelled.jpg]

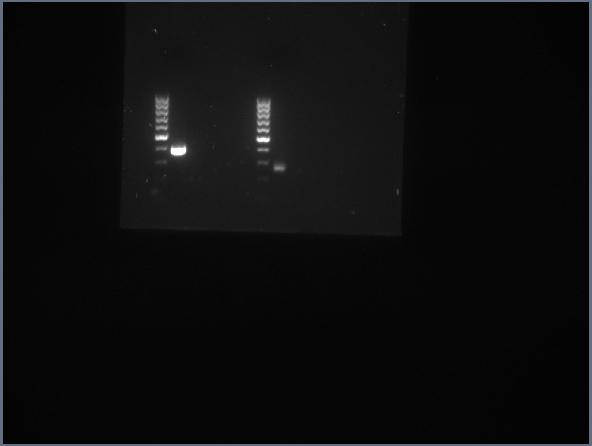

Supplement: Figure 7—source data 1. [file elife-83486-fig7-data1.zip › Figure 7-source data 1/Figure 7A-VEGFA gel original.jpg]

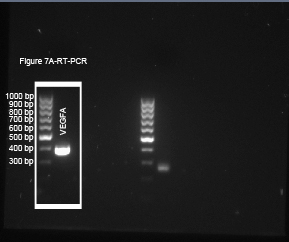

Supplement: Figure 7—source data 1. [file elife-83486-fig7-data1.zip › Figure 7-source data 1/Figure 7A-VEGFA gel labelled.jpg]

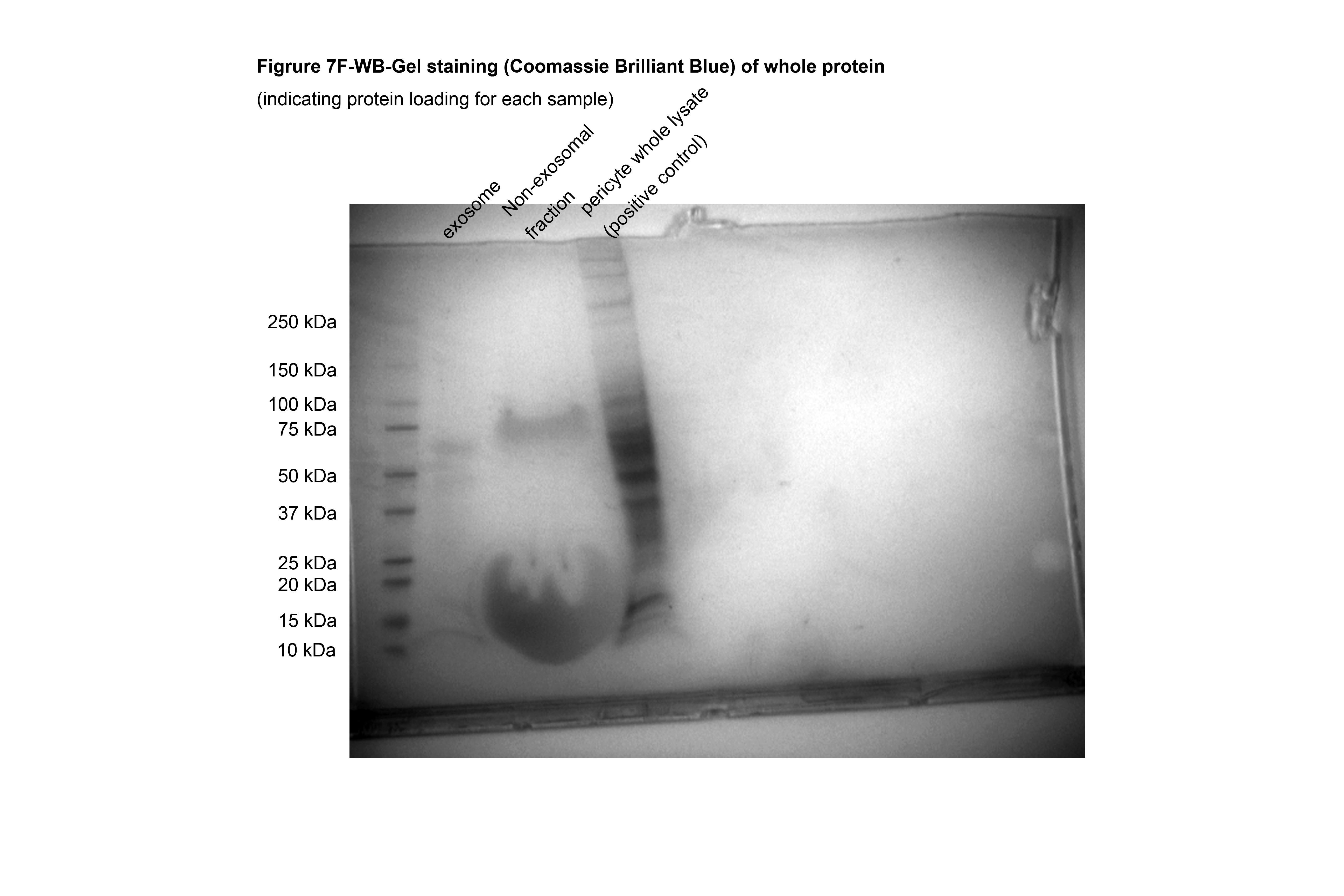

Supplement: Figure 7—source data 1. [file elife-83486-fig7-data1.zip › Figure 7-source data 1/Figure 7F-whole protein gel labelled.jpg]

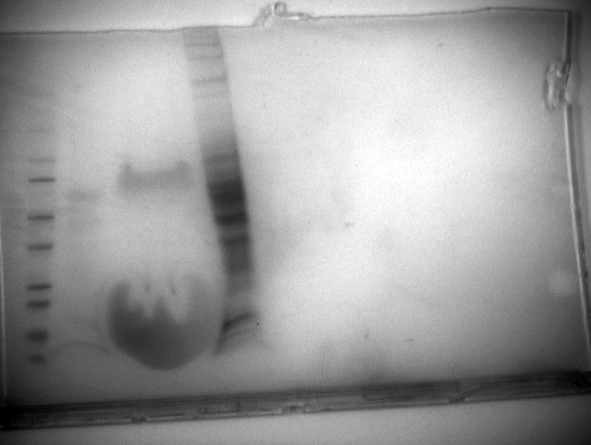

Supplement: Figure 7—source data 1. [file elife-83486-fig7-data1.zip › Figure 7-source data 1/Figure 7F-whole protein gel original.jpg]

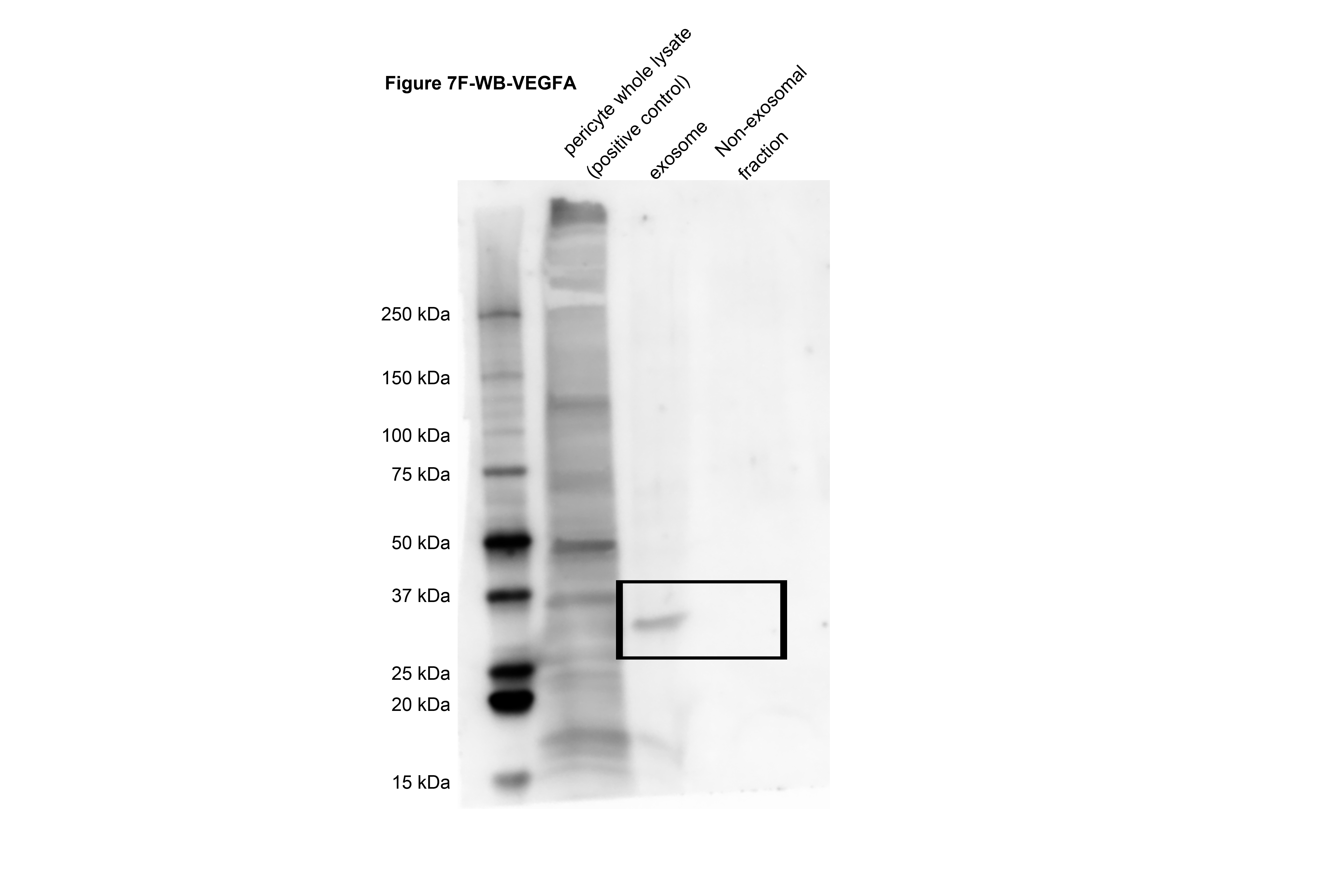

Supplement: Figure 7—source data 1. [file elife-83486-fig7-data1.zip › Figure 7-source data 1/Figure 7F-VEGFA blot labelled.jpg]

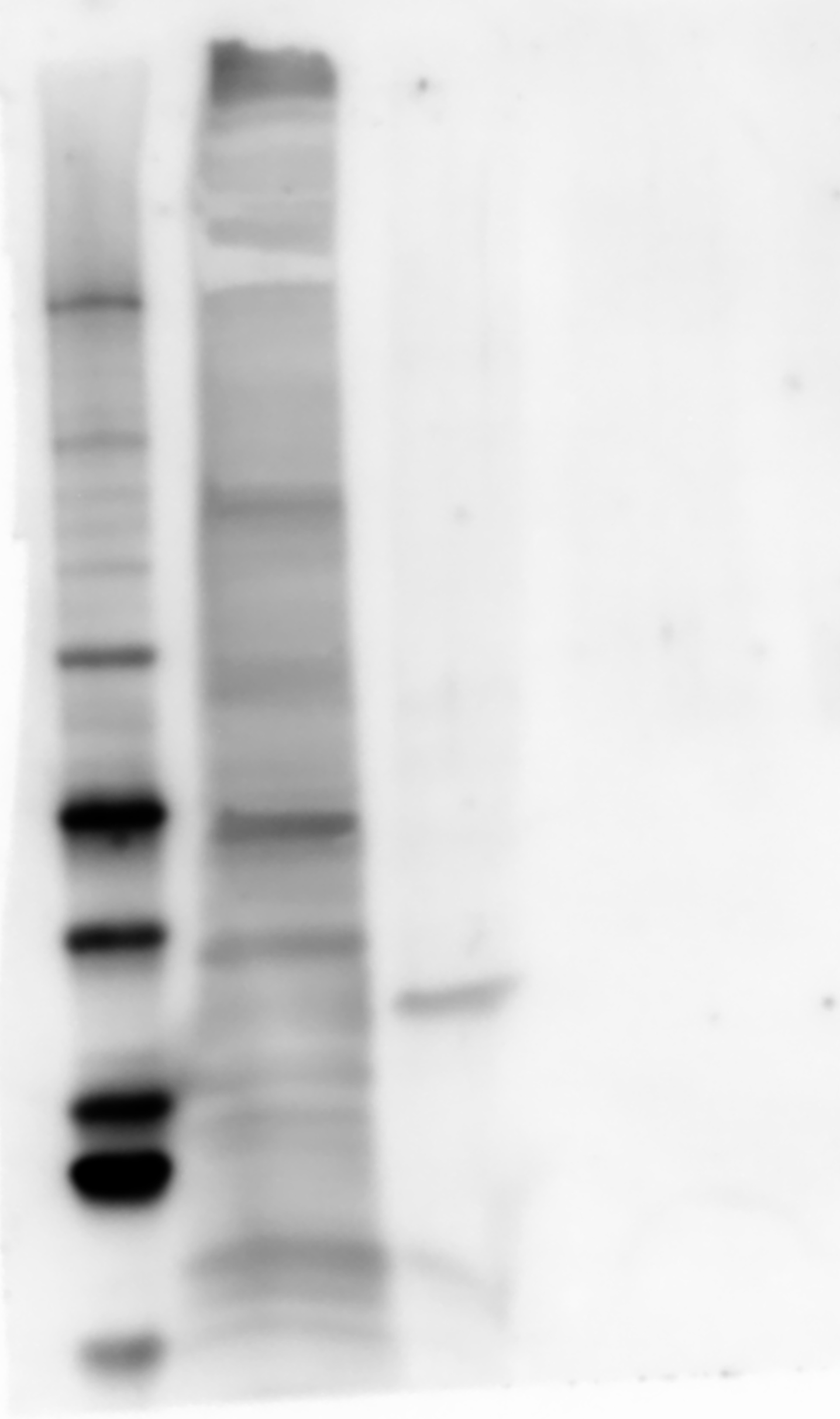

Supplement: Figure 7—source data 1. [file elife-83486-fig7-data1.zip › Figure 7-source data 1/Figure 7F-VEGFA blot original.jpg]

**VEGFA**

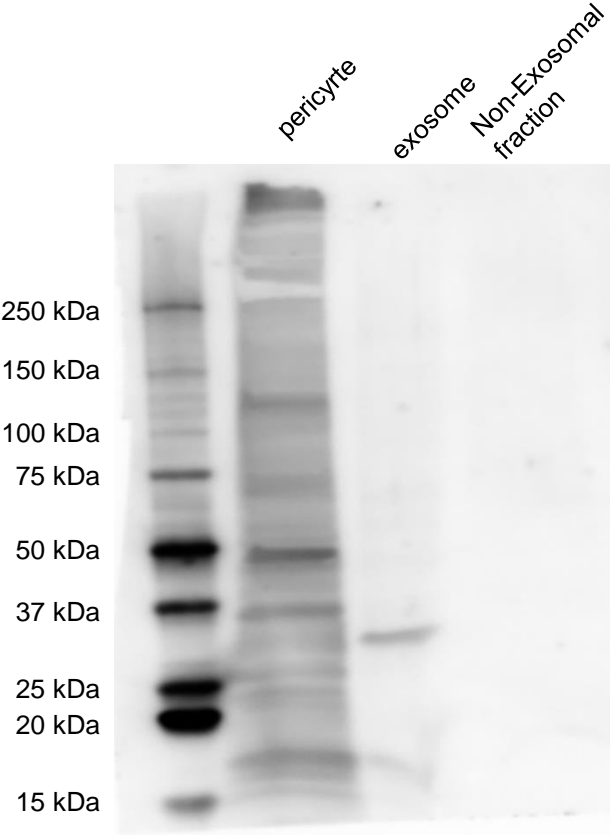

**PDGFR-β**

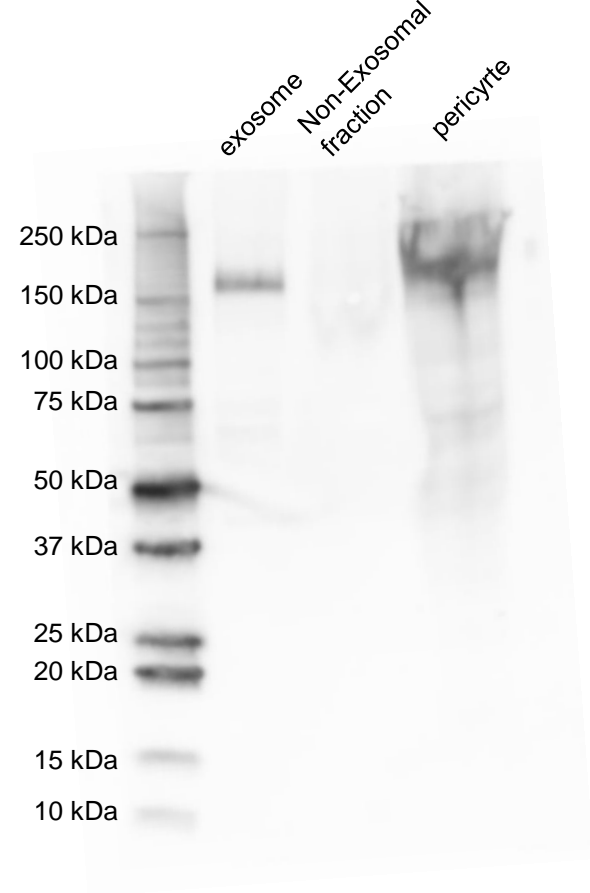

**Gel staining of whole protein**

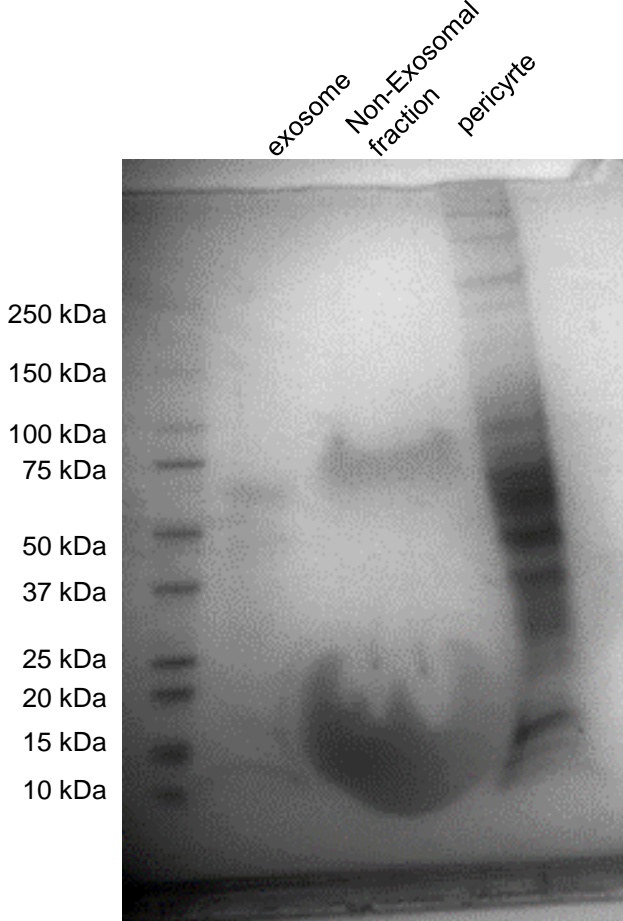

Supplement: Figure 7—source data 2. [file elife-83486-fig7-data2.pdf]
